# Preparation of the Yellow-Colored Aluminum Pigments with Double-Layer Structure Using a Crosslinked Copolymeric Dye

**DOI:** 10.3390/polym10101097

**Published:** 2018-10-04

**Authors:** Zhicheng Wang, Shuping Nie, Ru Xia, Yang Lv, Ming Cao, Ming Chen, Qiannian Dong, Lifen Su, Peng Chen, Bin Yang, Jibin Miao, Zhengzhi Zheng, Jiasheng Qian

**Affiliations:** 1 Key Laboratory of Environment-Friendly Polymeric Materials of Anhui Province, School of Chemistry and Chemical Engineering, Anhui University, Hefei 230601, China; honestwzc@163.com (Z.W.); spnam@sina.com (S.N.); lightmisaya@sina.com (Y.L.); cm630309441@163.com (M.C.); ausulf@gmail.com (L.S.); chpecp@126.com (P.C.); beanyoung@163.com (B.Y.); lingxiaoyu1003@163.com (J.M.); 10006@ahu.edu.cn (Z.Z.); qianjsh@ahu.edu.cn (J.Q.); 2 Hefei Sunrise Aluminum Pigments Co. Ltd., Hefei 231131, China; dongqn2005@126.com

**Keywords:** aluminum pigments, crosslinked copolymeric dye, corrosion resistance

## Abstract

Colorization for fabricating aluminum pigments has broad application prospects in recent years. In this study, yellow-colored aluminum pigments with the double-layer structure Al@SiO_2_@PFMV were prepared using a sol-gel method. A crosslinked copolymeric dye (PFMV) was firstly synthesized by radical polymerization using vinyl triethoxysilane (VTES) and a small molecular dye (FGMAC) as monomers. Then, colored aluminum pigments were prepared by hydrolysis and condensation of the copolymers on the surface of aluminum pigments. SEM, AFM, FTIR, and XPS were used to characterize the surface morphology and chemical structure of the colored aluminum pigments. It was found that the colored aluminum pigments have a heterogeneous and smooth surface layer. The anticorrosion results showed that the colored aluminum pigments had better chemical stability with significantly improving corrosion resistance compared to raw aluminum pigments and Al@SiO_2_ with the single-layer coating. Chromatism analysis indicated that the lightness of Al@SiO_2_@PFMV pigments decreased slightly and the color changed from silver-gray to yellow.

## 1. Introduction

Aluminum pigments, a kind of ultrathin flaky aluminum powders, have been widely used in printing, painting and coating industry owing to their excellent metallic luster, covering capability, and “flip-flop” effect [1,2,3,4]. However, a severe problem is that aluminum can easy to react with water under the acid or alkali condition, which not only results in gloss decrease, but also affects the metallic luster, and even causes security risks [5,6,7,8]. Therefore, it is necessary to develop a method for improving the corrosion resistance of aluminum pigments on the surface.

Among various kinds of improving the anticorrosion properties of aluminum pigments, one method is to use anticorrosion inhibitor with special functional groups, which can adsorb on the surface of aluminum pigments, such as citric acid, aminophenols, vanillin, and so on [9,10,11,12]. Compared with adsorption of anticorrosion inhibitor, the other promising method is forming an inorganic layer or organic layer on the surface of aluminum pigments. For example, Kiehl firstly proposed the inorganic SiO_2_ encapsulation of aluminum pigments using tetraethoxysilane (TEOS) as a precursor with a sol-gel method [13]. Zhang et al. reported a coated aluminum pigments with TEOS using the similar method, the results showed that the corrosion protection factor can reach 99.3% [7]. Zhou et al. found that aluminum pigments coated with SiO_2_ and TiO_2_ with the double- layer structure to improve the dispersibility and anticorrosive properties [14]. Another encapsulated method is to modify the pigments using organic layer. Synthetic strategies including in situ polymerization or grafting polymer chains onto the aluminum pigments have been developed [4,15,16,17,18] and results in the formation of a dense layer on the surface of aluminum pigments. All these proposed methods have achieved significant progress for improving the corrosion resistance of aluminum pigments. However, it should be noted that inorganic coatings, such as SiO_2_ or TiO_2_, showed excellent stability but have poor compatibility with a polymeric matrix when using in painting or printing industry [19,20]. Organic coatings using polymers showed good compatibility but the chemical stability is not enough in corrosion medium compared to inorganic coatings, especially in some extreme environment [21]. Recently, the aluminum pigments were coated with hybrid layers, inferred as organic-inorganic mixed coating, which not only improved their anti-corrosion property, but also enhanced their dispersivity and compatibility [22,23].

In addition to the anticorrosion properties, colorization for fabricating aluminum pigments has broad application prospects and is strongly welcomed in recent years [24]. The colored aluminum pigments not only have metallic luster, but also have chromatic colors, which is not the case compared to raw aluminum pigments. Usually, depositing a layer of metal oxides on the surface of aluminum pigments is most often used to prepare colorful aluminum pigments, such as iron oxide and cobalt oxide [25,26]. Colored aluminum pigments were prepared by this method have the characteristics of good chemical resistance and weather resistance. However, it is difficult to achieve vivid color using this method. In addition, their compatibility with polymeric matrix is poor when in use in painting or printing industry. To solve these problems, depositing organic pigments onto the surface of aluminum pigments with the aid of polymer can not only bring bright colors, but also improve their anti-corrosion properties and compatibility. For example, Fu et al. prepared a polymer encapsulated colorful aluminum pigment by an in-situ polymerization method, which was stable in strong alkaline or acidic conditions with good near-infrared reflectance [27]. A colored polymer/aluminum hybrid pigment was synthesized by copolymerizing with *n*-butyl acrylate and styrene on the surface of aluminum pigments. They found that both the length of the grafted chains and the dye/styrene molar ratio influenced the color of the resultant hybrid material [28].

**Scheme 1:**
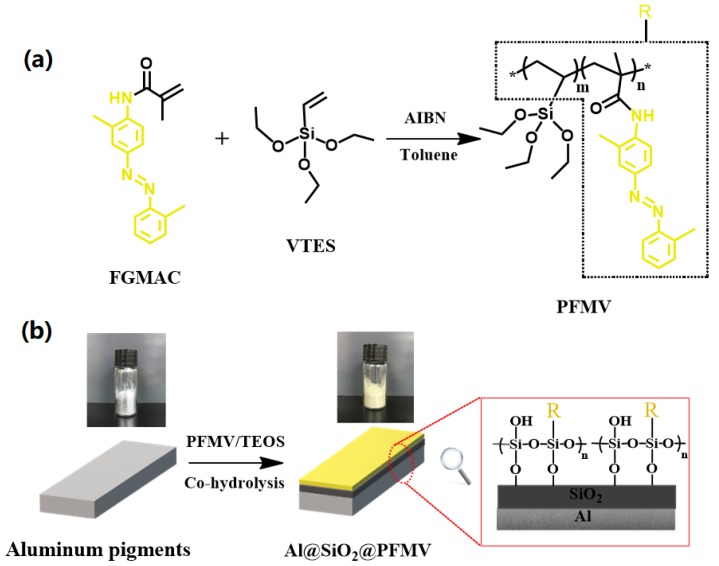
Schematic illustration of the preparation of PFMV (**a**) and yellow-colored Al@SiO_2_@PFMV pigments with double-layer structure (**b**).

In this paper, the main object is to introduce a method for the synthesis of colored aluminum pigments with the double-layer structure using a cross-linked copolymeric dye. It is expected that combination of the organic-inorganic mixed coating with introducing the coloring dyes, may produce colored aluminum pigments with better anti-corrosion properties. To obtain the desired effect, we firstly synthesized a new kind of copolymeric dye by radical polymerization using VTES and a small molecular dye FGMAC as monomers (Scheme 1a). Secondly, the colored aluminum pigments were prepared by hydrolysis and condensation of the copolymers on the surface of aluminum pigments (Scheme 1b). This method for preparation of the colored aluminum pigments has the following merits: (1) The dyes were introduced by covalent chemical bonds to achieve the purpose of coloring, which reduces the color fading over a long period of time compared to physical adsorption. (2) The double-layer structure will offer sufficient protection, reducing the corrosion on the surface of aluminum pigments. The chemical structure of the PFMV was analyzed. The surface morphology and chemical structure of the modified aluminum pigments were characterized by SEM, AFM, FTIR, and XPS. The anticorrosion properties and color performance of the prepared Al@SiO_2_@PFMV pigments were also investigated.

## 2. Materials and Methods

### 2.1. Materials

Aluminum pigments were purchased from Hefei Sunrise Aluminum Pigments Co., Ltd. (Hefei, China). Azobisisobutyronitrile (AIBN), tetraethoxysilane (TEOS), 4-amino-2′,3-dimethylazobenzene (FG), methacryloyl chloride (MAC), vinyltriethoxysilane (VTES), andbutylatedhydroxytoluene (BHT) were provided by Aladdin Co. Ltd., Shanghai, China. All other chemicals were purchased from Sinopharm Chemical Reagent Co., Ltd. (Shanghai, China), and used without further purification.

### 2.2. Preparation of FGMAC

The FGMAC was prepared according to the procedure described in the literature with slight modification [29]. 3.21 g of FG, 2.07 g of Et_3_N and 0.015 g of BHT were dissolved in 36 mL ether at 0 °C. 2.82 g of MAC was added dropwise into the solution under nitrogen atmosphere. The solution was stirred at room temperature overnight. The precipitate was filtered and the filtrate is dissolved in chloroform. The solution was washed with 5% HCl, water, 1 mol/L NaOH, and distilled water. The organic phase was dried with sodium sulfate and then the solvent was evaporated. The product was recrystallized from dichloromethane/hexane (1/3) with an orange solid FGMAC (yield 58%).

^1^H NMR (CDCl_3_): δ = 2.12 ppm (s, 3H, =C–C**H_3_**), 2.40 ppm (s, 3H, C**H_3_** ortho to –HN–C=O), 2.72 ppm (s, 3H, –C**H_3_** ortho to N=N), 5.53 ppm and 5.88 ppm (2s, 2H, –C=C**H_2_**), 7.33–7.54 ppm (m, 3H, aromatic **H** protons), 7.61–7.86 ppm (m, 4H, aromatic **H** protons ortho to N=N and HN–C=O), and 8.28 ppm (d, 1H, –**H**N–C=O).

### 2.3. Preparation of PFMV

0.586 g of FGMAC, 3.8 g of VTES and 0.044 g of AIBN were dissolved in 6 mL toluene. The solution was placed into a three-necked flask under nitrogen atmosphere. After three freeze-pump-thaw cycles, the above solution was heated at 70 °C for 48 h. After cooling to room temperature, the reaction mixture was poured into 200 mL ether. The precipitate was isolated, redissolved in chloroform, and purified by reprecipitation twice in the ether. The products were dried in a vacuum oven at room temperature for 24 h, yielding a red-brown solid at 83%.

^1^H NMR (CDCl_3_): δ = 1.26 ppm (–CH_2_C**H_3_**, C**H_3_**–C and –C**H_2_**–CH–Si–). 1.55 ppm (–C**H_2_**CCH_3_), 1.63–1.73 ppm (–CH_2_C**H**–Si–), 2.40 ppm (C**H_3_** ortho to –HN–C=O), 2.72 ppm (C**H_3_** ortho to N=N), 3.73 ppm (C***H*_3_**CH_2_-O), 7.33 ppm (aromatic **H** protons), 7.52 ppm (aromatic **H** proton ortho to N=N and CH_3_), 7.60–7.62 ppm (aromatic **H** proton ortho to N=N), 7.79 ppm (aromatic ***H*** proton ortho to NH), 7.83–7.85 ppm (aromatic ***H*** proton ortho to N=N), and 8.26–8.29 ppm (–N**H**–).

### 2.4. Preparation of Al@SiO_2_@PFMV

2.0 g of aluminum pigments were dispersed in 60 mL ethanol, and then ultrasonicated for 10 min to obtain the dispersed suspension. The suspension was transferred into a three-necked flask. Afterward, the prepared solution A (3 g PFMV, 2 g TEOS and 30 mL absolute ethanol) and solution B (3.0 g ammonia, 5 mL distilled water and 30 mL absolute ethanol) were added dropwise to the above solution at the same time. The mixture was stirred at 50 °C for 6 h. When the reaction was over, the dispersion was centrifuged to obtain the precipitate. The precipitate was washed by absolute ethanol for three times, dried in a vacuum oven at 60 °C for 24 h to obtain the Al@SiO_2_@PFMV composites. Al@SiO_2_ was prepared using the same process with 2 g of TEOS as the precursor.

### 2.5. Characterization

The ^1^H NMR spectra was carried out on a Bruker AVANCE III (Billerica, MA, USA) at 400 MHz (^1^H) using CDCl_3_ as a solvent. The molecular weight and polydispersity of polymers were measured by GPC (PL-120, Agilent Technologies, Santa Clara, CA, USA). Polystyrenes (PS) were used for the calibration with THF as the eluent (1 mL/min). The chemical structures of the aluminum pigments were analyzed by the Fourier transform infrared spectroscopy (FTIR) (NEXUS-870, Nicolet, Madison, WI, USA) scanning from 4000 to 500 cm^−1^. X-ray photoelectron spectroscopy (XPS, VG, Altrincham, UK) analysis was carried out on a ESCALAB-MK-II spectrometer equipped with AlKa X-ray source, operated at 150 W. Scanning electron microscope (SEM, S-4800, Hitachi, Japan) was used to observe the morphologies of the modified aluminum pigments. The surface roughness of the aluminum pigments was characterized by Atomic force microscope (AFM, D3000, Shanghai Zhuolun MicroNano Equipment Co., Ltd., Shanghai, China). Thermo degradation was detected by a thermo gravimetric analyzer (Pyris-1, PerkinElmer, Waltham, MA, USA) under nitrogen atmosphere ranging from 50 to 800 °C at a heating rate of 20 °C/min. The color performance of the pigments was analyzed by an MA-94 color analyzer (X-Rite, Grand Rapids, MI, USA). The particle size was analyzed using a S3500 analyzer (Microtrac, Montgomeryville, PA, USA). Corrosion resistance test was accomplished using the method described in the literature [16], by placing 0.1 g aluminum pigments in pH 1.0 hydrochloric acid or pH 12 sodium hydroxide solution for 168 h according to the quantity of hydrogen.

## 3. Results and Discussion

Colored aluminum pigments with the double-layer structure Al@SiO_2_@PFMV were prepared by a two-step process. Firstly, PFMV was synthesized by radical copolymerization using VTES and FGMAC as monomers; the products could be easily separated by precipitation in ethyl ether, obtained in 83% yield. Secondly, the PFMV was introduced to the aluminum surface co-hydrolyzed with TEOS as precursors. Due to a large number of hydroxyl groups present on the surface of aluminum pigments, the ethoxy groups of TEOS and PFMV could be hydrolyzed and condensed on the surface of aluminum pigments using aqueous ammonia as a catalyst (Scheme 1).

### 3.1. Preparation and Characterization of PFMV

**Figure 1:**
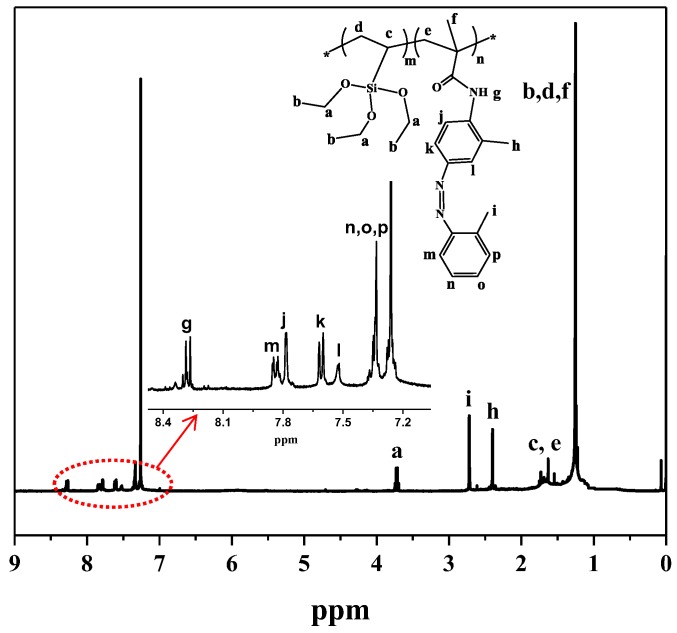
^1^H NMR spectra of PFMV.

**Figure 2:**
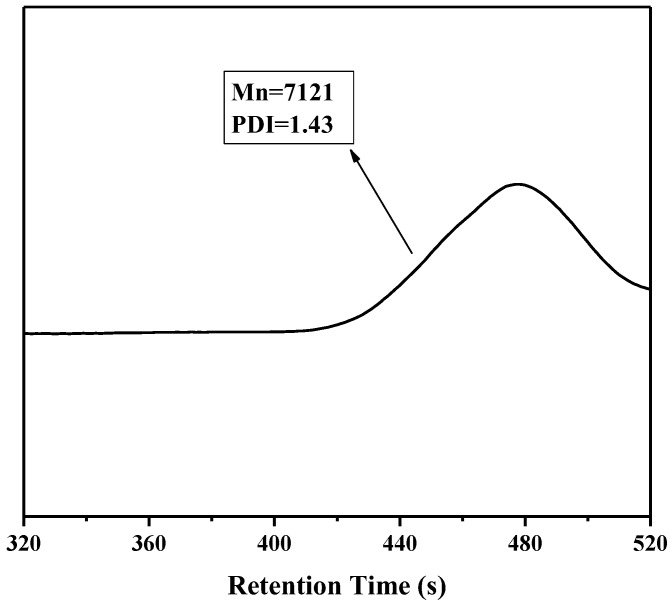
GPC spectra of PFMV.

The structure of the PFMV copolymers was characterized by ^1^H NMR. As shown in Figure 1. The appearance of the new peaks both from FGMAC and VTES, and the disappearance of peaks (C**H**_2_=C**H**–) around 5.53 and 5.88 ppm attributed to groups from FGMAC and 5.3 ppm ascribe to the C**H**_2_= groups from VTES. The molar ratio of VTES to FGMAC was 1/4, which can be calculated by the integral ratio of (3.71–3.73 ppm, –C**H**_2_–O) in the VTES blocks and (2.40 ppm, C**H**_3_ ortho to N–C=O) in FGMAC blocks. The copolymers were further characterized by GPC with polystyrene as standards. As shown in Figure 2, the GPC curve presented a unimodal distribution, good symmetry, and no tail phenomenon. The *M*_n_ of the copolymers is about 7121 Da and the PDI (*M*_w_/*M*_n_) is 1.43.

**Figure 3:**
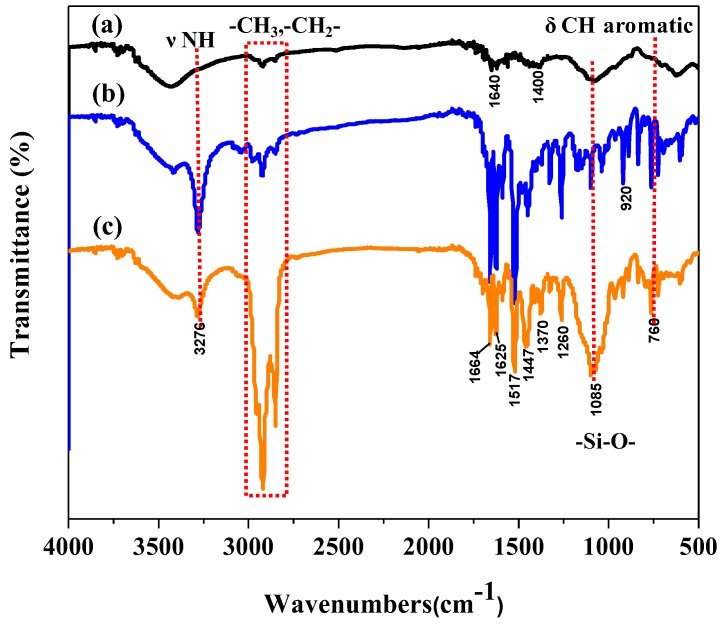
FTIR spectra of VTES (**a**), FGMAC (**b**) and PFMV (**c**).

Figure 3 showed the FTIR spectra of VTES (curve a), FGMAC (curve b) and PFMV (curve c). The spectrum of the VTES (curve a) exhibited characteristic absorptions of Si–O–R at 1085 cm^−1^, C=C at 1640 cm^−1^and δ CH_3_ at 1400 cm^−1^ [19]. The spectrum of the FGMAC (curve b) exhibits characteristic peaks at 3276 cm^−1^ (ν NH), 1664 cm^−1^ (ν C=O amide I), 1625 cm^−1^ (δ NH), 1517 cm^−1^ (ν C=C aromatic), 1447–1370 cm^−1^ (δ CH_3_), 1260 cm^−1^ (ν C–N), 920 cm^−1^ (δ CH=C), and 887–760 cm^−1^ (δ CH aromatic) [29]. For PFMV (curve c), there are clearly absorptions at 3276 cm^−1^ of NH, 1085 cm^−1^ of Si–O–R and many characteristic peaks which showed in curve (b), and the disappearance of peaks around 1640 cm^−1^ and 920 cm^−1^ attributed to C=C groups. All these results indicated that PFMV was synthesized successfully.

### 3.2. Preparation and Characterization of the Al@SiO_2_@PFMV

#### 3.2.1. FTIR Analysis

**Figure 4:**
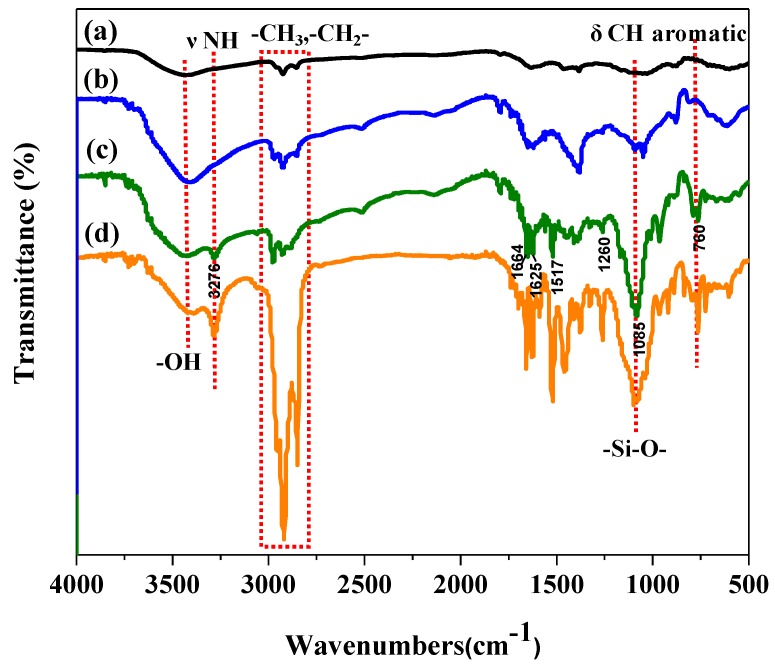
FTIR spectra of raw aluminum pigments (**a**), Al@SiO_2_ (**b**), Al@SiO_2_@PFMV (**c**), and PFMV (**d**).

Figure 4 showed the FTIR spectra of raw aluminum pigments (curve a), Al@SiO_2_ (curve b), Al@SiO_2_@PFMV (curve c), and PFMV (curve d). In curve (a), the absorption peaks at approximately 3400 cm^−1^ can be attributed to the -OH groups. For Al@SiO_2_, aluminum pigment modified with pure TEOS exhibited new characteristic absorptions of Si–O–Si at 1050 cm^−1^ [19]. For Al@SiO_2_@PFMV, the characteristic absorptions of Si–O–Si at 1085 cm^−1^, ν NH at 3276 cm^−1^, ν C=O amide I at 1664 cm^−1^, δ NH at 1625 cm^−1^, ν C–N at 1260 cm^−1^, benzene of ν C=C aromatic at 1517 cm^−1^, and δ CH aromatic at 760 cm^−1^ were observed, which suggested that the hydroxyl groups of the aluminum pigments underwent condensation reactions with PFMV and TEOS to form inorganic-organic layer.

#### 3.2.2. XPS Analysis

**Figure 5:**
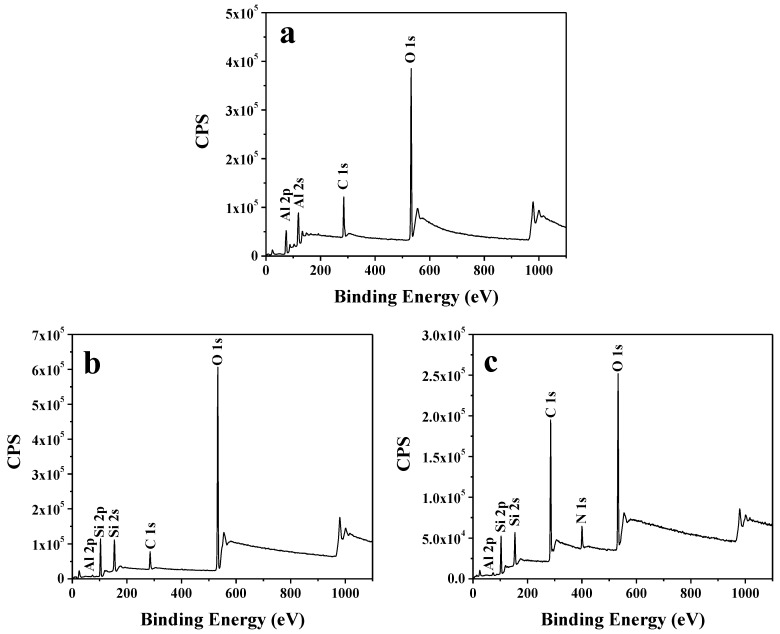
XPS spectra of raw aluminum pigments (**a**); Al@SiO_2_ (**b**); and Al@SiO_2_@PFMV (**c**).

To further characterize the modified aluminum pigments, surface chemical changes and chemical compositions were analyzed by XPS. The elemental contents of three samples including raw aluminum pigments, Al@SiO_2_ and Al@SiO_2_@PFMV were listed in Table 1. As shown in Figure 5a, the characteristic peaks of Al2p can be found at approximately 71.9 eV. The O1s and C1s elements at 285.1 and 536.3 eV can also be observed, which could be attributed to the residual higher fatty acid or solvent oil used in the manufacturing process of aluminum pigments. After being modified with TEOS, the spectra showed new peaks at binding energy of 103.1 and 151.5 eV (Figure 5b) [30], which were ascribed to Si2p and Si2s. The content of Al element decreased from 49.78% to 3.93% and the C element decreased from 25.4% to 12.25%. For Al@SiO_2_@PFMV, with the exception of the Si2p and Si2s (103.1 and 151.5 eV) peaks, the new element N (N 1s, 400.1 eV) appeared (Figure 5c) [23]. In addition, the content of Al2p sharply decreased to 3.74%, and C1s increased from 25.4% to 40.02%, suggested that TEOS and PFMV have been successfully coated on the surface of aluminum pigments and formed a relative denser encapsulation than TEOS.

**Figure 6:**
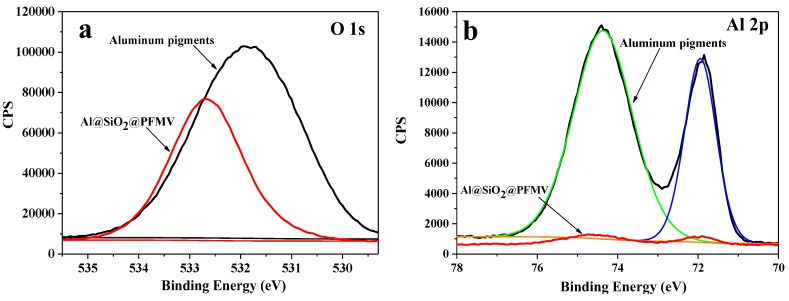
High-resolution XPS spectrum of O 1s (**a**) and Al 2p (**b**).

The surface chemical changes of the samples were further conducted to elucidate the chemical bonds, which were fitted using the software XPSPEAK. The high-resolution O1s spectra of Al@SiO_2_@PFMV was shown in Figure 6a, it was found that the O1s peak shifted from 531.6 eV to 532.9 eV [23], suggesting that the chemical environment changed after the coating process. As shown in Figure 6b, the spectra of raw aluminum pigments showed a double peak, the Al2p peak at 71.9 eV and 74.5 eV was attributable to aluminum and Al_2_O_3_, respectively. After being modified with PFMV, the Al2p peak shifted from 74.5 to 75.0 eV, suggesting that the chemical environment corresponding to the Al–O bond changed. This chemical shift indicated that new Al–O–Si bonds were formed on the aluminum surface after the coating [19].

#### 3.2.3. Particle Size Analysis

**Figure 7:**
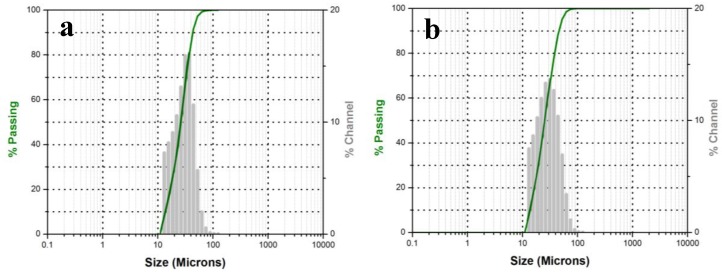
Particle size distributions of (**a**) raw aluminum pigments and (**b**) colored aluminum pigments.

To verify the size change of the aluminum pigments after the encapsulation, the particle size distribution was analyzed. The average diameter (*d*_50_) of colored aluminum pigments is about 28.23 μm (Figure 7a), which is slightly larger than that of raw aluminum pigments with the size of 27.98 μm (Figure 7b). The size change ascribed to the formed thin films on the surface of the raw aluminum pigments.

#### 3.2.4. Morphologies of the Aluminum Pigments

**Figure 8:**
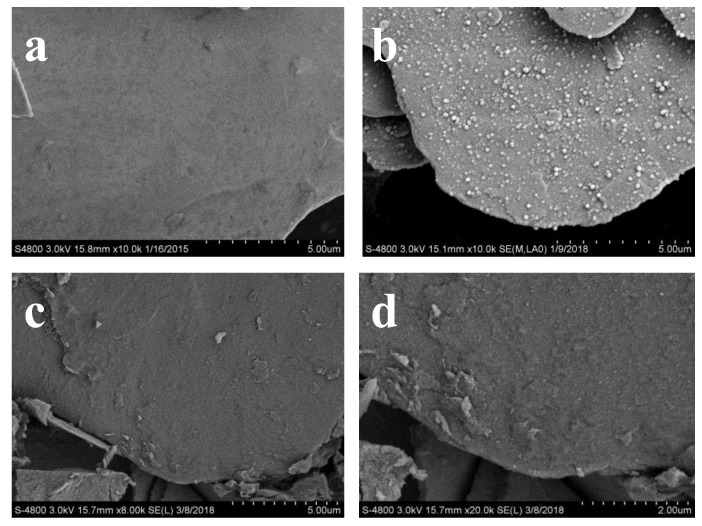
SEM images of raw aluminum pigments (**a**); Al@SiO_2_ (**b**); and Al@SiO_2_@PFMV (**c**,**d**).

SEM was carried out for analyzing the surface morphology of aluminum pigments. Figure 8a indicated the raw aluminum pigments showed a smooth flaky powder. Figure 8b showed the aluminum pigments were encapsulated by TEOS. PFMV co-hydrolyzed with TEOS was shown in Figure 8c (8k amplified factor) and Figure 8 (20k amplified factor). As can be seen from the Figure 8b, the SiO_2_ particles were uniformly distributed on the surface of aluminum pigment with size about 100 nm. In contrast, we can find that the aluminum pigments were encapsulated in a dense film using PFMV and TEOS to form the inorganic-organic layer (Figure 8c,d). The surface of the aluminum pigments, which were coated by PFMV and TEOS, was more smooth than that of Al@SiO_2_ in Figure 8b.

**Figure 9:**
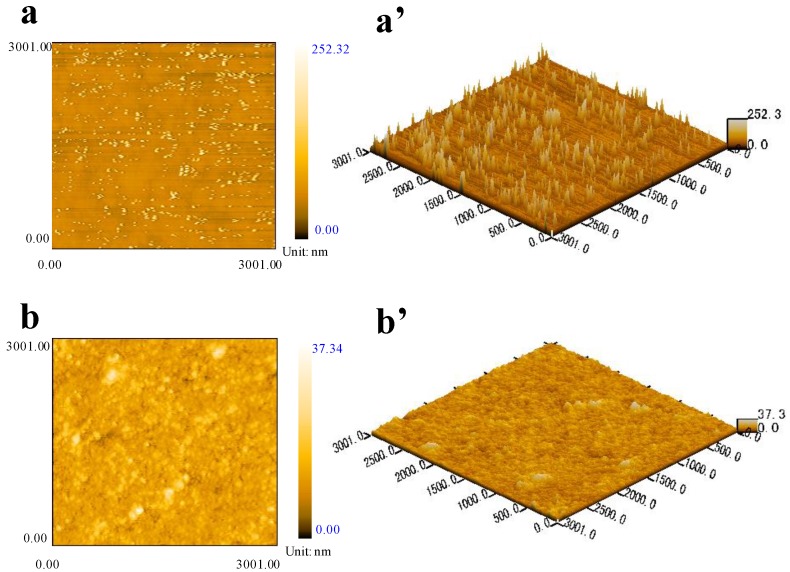
The AFM images of Al@SiO_2_ (**a**,**a′**) and Al@SiO_2_@PFMV (**b**,**b′**).

AFM was used to characterize surface roughness of the Al@SiO_2_ and Al@SiO_2_@PFMV. As shown in Figure 9a,a′, it is obvious that there are many SiO_2_ particles formed on the surface of aluminum pigments, the maximum and average roughness of SiO_2_ particles were about 233.6 nm and 9.52 nm, respectively. In contrast, the surface of the Al@SiO_2_@PFMV pigments was much smoother (Figure 9b,b′). The maximum and average roughness were about 33.3 nm and 1.62 nm, respectively. The topographical parameters, including the root mean square of the surface (*R*_q_), the average roughness (*R*_a_), and the maximum roughness (*R*_max_), were shown in Table 2. These results were consistent with the SEM results.

#### 3.2.5. TG Analysis

**Figure 10:**
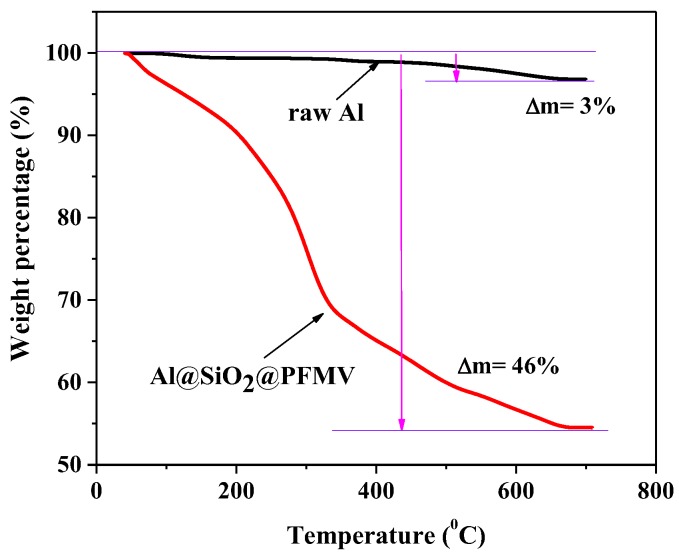
TG analysis of raw Al pigments and Al@SiO_2_@PFMV.

Figure 10 showed the results of TG characterization of raw aluminum pigments and modified aluminum pigments. The quality of raw aluminum pigments decreased gradually with the increase of temperature range, which could be attributed to the decomposition of residual higher fatty acid or solvent oil used in the manufacturing process of aluminum pigments. The total weight loss values were about 3.0%. For Al@SiO_2_@PFMV, it was observed that the samples showed a multi-stage decomposition, and had a much faster decomposition rate, which may account for the PFMV decomposition on the surface of aluminum pigments. The total weight loss values of about 46%, suggested that the PFMV have been hydrolyzed and condensed on the surface of aluminum pigments.

### 3.3. Corrosion Resistance Tests

**Figure 11:**
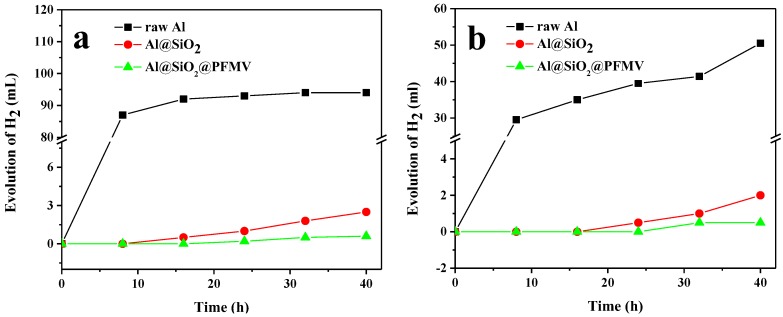
Corrosion resistance of raw aluminum pigments, Al@SiO_2_ and Al@SiO_2_@PFMV at pH 1.0 (**a**) and at pH 12 (**b**).

**Figure 12:**
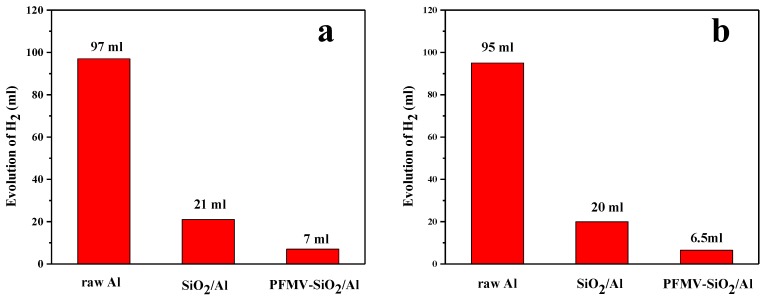
The volume of H_2_ evolved from the raw aluminum pigments, Al@SiO_2_ and Al@SiO_2_@PFMV pigments within 168 h at pH 1.0 (**a**) and at pH 12 (**b**).

To verify the combination of organic-inorganic mixed coating with the double-layer structure may bring better anticorrosion properties. The corrosion resistance of the raw aluminum pigments, Al@SiO_2_ and Al@SiO_2_@PFMV were quantified by collecting the H_2_ gas at different time under pH 1.0 and pH 12. Clearly, the modified aluminum pigments have better stability than raw aluminum pigments. As shown in Figure 11, the Al@SiO_2_ and Al@SiO_2_@PFMV pigments in the former 15 h can maintain good corrosion resistance in strong alkaline or acidic conditions. Both the Al@SiO_2_ and Al@SiO_2_@PFMV formed a dense coating layer could prevent Al from reacting with the corrosive medium. After 20 h, the collected gas volume from Al@SiO_2_ is obviously higher than that of Al@SiO_2_@PFMV. We further extended the test time to 168 h, as shown in Figure 12, the volume of H_2_ evolved from the Al@SiO_2_@PFMV pigments was about one third that from Al@SiO_2_ at pH 1.0 and pH 12. These results indicated that the protection efficiency of Al@SiO_2_@PFMV with the double-layer structure was more efficient than the Al@SiO_2_ with the single-layer coating. The grafting of the PFMV could further improve the corrosion resistance of Al@SiO_2_ under the acid or alkali condition.

### 3.4. Chromatism Analysis

CIE L*a*b* is an important parameter to analyze the visible light optical properties, defined by the International Commission on Illumination. It expresses color as three values, L* for the lightness, a*, and b* for the green-red and blue-yellow color components. The CIE L*a*b* values of raw aluminum pigments, Al@SiO_2_ and Al@SiO_2_@PFMV were analyzed by the color analyzer. The results were listed in Table 3. When the test angle is 15°, the lightness of Al@SiO_2_ and Al@SiO_2_@PFMV slightly decreased compared to the raw aluminum pigments, which ascribe to that a dense film was formed on the surface of aluminum pigments. The values of a* and b* were −0.31 and −2.68 for aluminum pigments, respectively. For Al@SiO_2_@PFMV, the values of a* and b* were −1.61 and 14.04. The a* value is slightly decreased and the b* value increased a lot, indicated that the color of Al@SiO_2_@PFMV changed from silver-gray to yellow. The CIE L*a*b* values were also measured with varying angles. From Table 3, it can be found that the lightness of the pigments decreased with the increasing measuring angle for every sample. The a* and b* values for all the three sample changed little with the different angles.

## 4. Conclusions

In this study, a novel cross-linked copolymeric dye PFMV was prepared and characterized. Yellow-colored aluminum pigments with the double-layer structure Al@SiO_2_@PFMV were prepared by hydrolysis and condensation of PFMV and TEOS as precursor. The surface morphology and chemical structure of the colored aluminum pigments were characterized by SEM, AFM, and XPS. The PFMV and TEOS could form a dense and smooth inorganic-organic film coated at the surface of the raw aluminum pigments, which could not only improve their corrosion resistance, but also make the color change from silver-gray to yellow.

## Figures and Tables

**Table 1:** Elemental contents on the surface of aluminum pigments.

Sample	Elemental Contents (PP, at%)				
	Al	C	O	Si	N
raw Al	49.78	25.4	24.82	/	/
Al@SiO_2_	3.93	12.25	42.6	41.22	/
Al@SiO_2_@PFMV	3.74	40.02	24.83	24.38	7.03

**Table 2:** The topographical parameters for Al@SiO_2_ and Al@SiO_2_@PFMV.

Samples	*R*_q_ (nm)	*R*_a_ (nm)	*R*_max_ (nm)
Al@SiO_2_	9.64	9.52	233.6
Al@SiO_2_@PFMV	1.65	1.62	33.3

*R*_q_: the root mean square of the surface; *R*_a_: the average roughness; *R*_max_: the maximum roughness.

**Table 3:** The CIE L*a*b* values of aluminum pigments, Al@SiO_2_ and Al@SiO_2_@PFMV.

Samples		Aluminum Pigments	Al@SiO_2_	Al@SiO_2_@PFMV
15°	L*	159.3	136.86	111.6
	a*	−0.31	−0.18	−1.61
	b*	−2.68	−0.53	14.04
25°	L*	109.68	109.82	94.04
	a*	−1.03	−0.73	−2
	b*	−1.79	−0.27	15.03
45°	L*	52.35	67.04	68.64
	a*	−0.44	−0.38	−1.57
	b*	−1.19	−0.27	14.56
75°	L*	31.73	41.27	49.5
	a*	−0.52	−0.7	−1.34
	b*	−0.94	0.55	16.59
110°	L*	26.22	34.11	41.51
	a*	−0.6	−0.82	−1.12
	b*	−1.1	0.82	18.5
